# Implementation of a nurse-led self-management support intervention for patients with cancer-related pain: a cluster randomized phase-IV study with a stepped wedge design (EvANtiPain)

**DOI:** 10.1186/s12885-020-06729-0

**Published:** 2020-06-16

**Authors:** Silvia Raphaelis, Florian Frommlet, Hanna Mayer, Antje Koller

**Affiliations:** 1 Department of Nursing Science, University of Vienna, Alser Strasse 23/12, 1080 Vienna, Austria; 2 Center for Medical Statistics, Informatics and Intelligent Systems (Institute of Medical Statistics), Medical University of Vienna, Vienna, Austria; 3 Institute of Applied Nursing Science, University of Applied Sciences, St. Gallen, Switzerland

**Keywords:** Pain, Randomized controlled trials, Oncology nursing, Neoplasms, Patient education as topic, Self-management

## Abstract

**Background:**

Pain self-management support interventions were effective in controlled clinical trials and meta analyses. However, implementation of these complex interventions may not translate into identical effects. This paper evaluates the implementation of ANtiPain, a cancer pain self-management support intervention in routine clinical practice according to the **R**each **E**fficacy-**A**doption **I**mplementation **M**aintenance framework.

**Methods:**

In this cluster randomized study with a stepped wedge design, *N* = 153 adult patients with cancer-related pain were recruited from 01/17 to 05/18 on 17 wards of 3 hospitals in Vienna, Austria. ANtiPain entailed a face-to-face in-hospital session by a trained nurse to prepare discharge according to key strategies, information on pain self-management, and skills building. After discharge, cancer-pain self-management was coached via phone calls. Patient-level data were collected at recruitment, and 2, 4 and 8 weeks after discharge via postal or online questionnaire. Primary outcome was pain interference with daily activities. Secondary outcomes included pain intensity, self-efficacy, and patient satisfaction. Organizational-level data (e.g., on implementation procedures) were collected by study or intervention nurses. The mixed model to analyze patient-level data included a random intercept and a random slope for individual and a random intercept for ward.

**Results:**

Recruitment was slower than expected and unevenly distributed over wards and hospitals. The face-to-face session was clinically feasible (mean duration = 33 min) as well as the mean amount (*n* = 2) and duration of phone calls (mean = 17 min). Only 16 (46%) of 35 trained nurses performed the intervention on nine wards. To deal with the loss of power, analyses were adapted. Overall effects on pain interference were not significant. However, effects were significant in sub analyses of the nine wards that recruited patients in the intervention period (*p* = .009). Regarding secondary outcomes, the group-by-time effect was significant for self-efficacy (*p* = .033), and patient satisfaction with information on pain-self-management (*p* = .002) and in-hospital pain management (*p* = .018).

**Conclusions:**

The implementation of ANtiPain improved meaningful patient outcomes on wards that applied the intervention routinely. Our analyses showed that the implementation benefited from being embedded in larger scale projects to improve cancer pain management and that the selection of wards with a high percentage of oncology patients may be crucial.

**Trial registration:**

ClinicalTrials.gov Identifier: NCT02891785 Date of registration: September 8, 2016.

## Background

The gold standard to establish an intervention’s efficacy is still the randomized controlled trial. However, supposing that one can rollout thus tested interventions into real life clinical practice with only minor changes may be misleading. Instead, reality is a bit more complicated [1]. Implementation research promotes the translation of research findings into real-life clinical practice to improve healthcare and closes a well-known gap between bench and bedside science. It explores those challenges we face when transferring state-of-the-art research findings into the “real world” [2]. Implementation trials (e.g., phase IV, effectiveness or pragmatic trials) characteristically address various aspects of implementation, e.g. (a) factors affecting implementation, (b) processes of implementation, and (c) the results of implementation [2]. The purpose is to understand what, why, and how interventions work in real-life clinical settings and to test ways to improve them. Rather than trying to control for “real-world” conditions or to remove their influence, implementation research seeks to understand and work within these conditions [2].

Looking at pain self-management support interventions from this perspective, it becomes apparent, that they have shown to be effective in many clinical trials and quite a few meta-analyses [3–7]. This kind of interventions are so-called *complex health care interventions* because they consist of several interacting components that are highly sensitive to context (e.g. providers, receiver, or content) [8]. Despite this context sensitivity, only few studies explored so far what happens with effects when pain self-management interventions are implemented in real-life clinical practice.

### Significance

Unceasingly high rates of pain in oncology patients suggest that adequate pain control seems to remain a persistent problem in oncology care [9–11]. Considering the shift towards outpatient settings in oncology, patients themselves play a crucial role in their pain management [7]. Those things that may hinder patients to optimally self-manage their pain are so-called patients-related barriers towards pain management [12]. For instance, patients still fear pain medication-associated tolerance and addiction. Furthermore, they may lack skills and knowledge concerning effective pain self-management [12, 13].

The *PRO-Self© Plus Pain Control Program* (PCP) has been shown to be an effective self-management support intervention by reducing these patient-related barriers towards cancer pain management [3, 14]. The intervention was initially developed and successfully tested in the United States [14] and has then been translated, adapted, and tested in the German-speaking context with two pilot randomized controlled trials (RCT) [15, 16]. In the first pilot RCT in the German context (PEINCA, *n* = 39), patient-related barriers were significantly reduced (*p* = .04), whereas average and worst pain, opioid intake and self-efficacy remained unchanged when compared to standard care [15]. Furthermore, a nested qualitative sub-study showed that participants were highly satisfied with the program and that it helped them to deal with pain [17]. However, the structure of the intervention still did not correspond with clinical real-life conditions in the German-speaking context. For example, specialized home-based interventions for oncology patients are not ubiquitous in Germany or Austria and their coverage of the health care insurances is not clear. Therefore, to make it clinically more realistic, the structure of the intervention, now called *ANtiPain*, was further revised. In particular, ANtiPain now has a more adaptable structure so that it can follow standard clinical care more flexibly whilst core components of the original intervention were kept (e.g., the three key components information, nurse coaching and skills building) [18]. In the second German pilot RCT (*n* = 39), pain intensity, pain interference with daily activities, and pain-related self-efficacy reduced non-significantly with moderate to high effect sizes with the adapted ANtiPain intervention, while patient-related barriers to cancer pain management improved significantly (*p* = .03) [16].

Following the custom paradigm, most clinical trials evaluating pain self-management support interventions focus on the elimination of potential confounders and include specifically chosen participants and settings as homogeneous and motivated as possible [3, 7]. While this approach gives important information on the efficacy and internal validity of an intervention, it may result in expensive and demanding programs that are difficult to implement in the real-world of clinical practice [19]. The process of implementation includes a course of bargaining expenses and efforts that may be spent for implementation in the settings as well as taking clinicans’ and patients’ preferences into account [20, 21]. Because effectiveness research (in contrast to efficacy research) is complex by nature and usually combines multiple evaluation research methods to depict a broader picture of effectiveness in clinical reality, the view on what is accepted as “proof” differs from that usually taken in custom RCTs. This approach does not mean that evaluation research asks for less rigid levels of quality measures. Instead, it takes a broader approach using different sources of information by which effects may be attributed to the implementation of the intervention in question, accounting for processes and changes that may be met in routine clinical practice [2].

A framework that has been developed to guide implementation and its evaluation in a research context is the Reach Efficacy Adoption Implementation and Maintenance (RE-AIM) framework [19, 22]. In this theoretical approach, (1) **R**each refers to the external validity of the study, i.e., whether the participants of the study are qualitatively and quantitatively in congruence with the target population. (2) **E**fficacy or **E**ffectiveness refers to the extent to which the targeted behavioral outcome can be achieved when the intervention is implemented as intended. While efficacy can be defined as the performance of an intervention under quite controlled conditions, effectiveness refers to its performance in the ‘real clinical world’. In this study, we aim at establishing the effectiveness of ANtiPain in the context of the RE-AIM framework. (3) **A**doption refers to the likelihood that an intervention is implemented by targeted institutions. (4) **I**mplementation refers to the extent to which the intervention is performed as intended in the real clinical setting. (5) **M**aintenance describes the extent to which the intervention and its effects will be sustained by patients as well as by the applying institutions. While the first two domains are usually evaluated on the individual level, Adoption and Implementation are evaluated on the organizational level. Last, the Maintenance domain is viewed from both, the individual patient as well as the organizational level [22]. Therefore, the aim of this paper is to report on the evaluation of the implementation of the ANtiPain self-management intervention in a realistic German-speaking setting on the domains of **R**each, **E**ffectiveness and **I**mplementation. Results will not only be useful for the evaluation of the interventions’ effectiveness but will also yield important information for institutions that pursue the improvement of pain management.

To our knowledge, this is the first study to comprehensively evaluate the implementation of a cancer pain self-management support intervention in routine clinical practice according to the RE-AIM framework. In this paper, we will mainly report on patient-related outcomes from the first two domains of the RE-AIM framework, namely **R**each and **E**ffectiveness. Specific aims of the study were to (1) describe recruitment and characteristics of the target population (**R**each); (2) to report on overall effectiveness of the intervention (**E**ffectiveness) and (3) which elements of implementation may play a role on the effectiveness of the intervention (**I**mplementation). **A**doption of the intervention will be reported elsewhere, and **M**aintenance of the intervention may be addressed in a second study and was beyond the scope of this evaluation study.

## Methods

### Study design

**Fig. 1:**
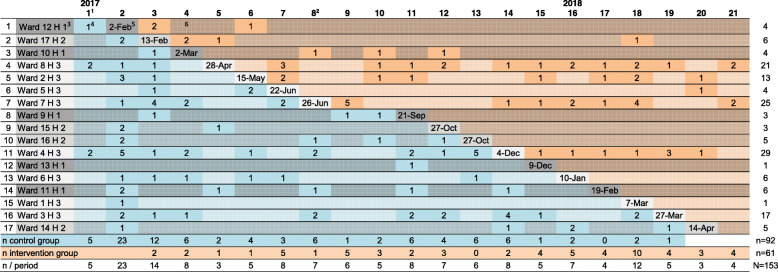
Stepped wedge plan and recruitment during 17-months study period. ^1^the columns represent the study periods of the stepped wedge study, one study period was 24 days (data collection from Jan 2017 to May 2018). ^2^During the summer, recruitment was paused for two periods. ^3^H: hospital; H1 shaded dark grey, H2 shaded lighter grey, H3 shaded lightest grey; the order of the implementation was randomized over all three settings. ^4^Number of recruited patients in the respective cell (time period on the respective ward). ^5^Date of actual ANtiPain training for intervention nurses on respective ward, steps were planned every 24 days. ^6^Shaded areas indicate that no patient was recruited on that ward during that time

This study (EvANtiPain) was planned as a cluster randomized phase-IV study (cRCT) with a stepped wedge design (clinical trial ID: NCT02891785). A phase-IV study was conducted to finally evaluate the implementation of ANtiPain in routine practice [21]. The stepped wedge design was selected because it allowed a sequential intervention rollout with corresponding before and after measures in each cluster given that recruitment is evenly distributed over time (Fig. 1) [23]. Thus, the intervention was implemented at certain randomized time steps at which one ward (cluster) after another changed from control to intervention condition until each had implemented ANtiPain (Fig. 1) [24]. For the stepped wedge design, the sequence of implementation was randomized. Randomization was performed on ward level.

### Interventions

In this study, implementation is viewed as a social process that is inseparable from the setting in which it takes place. **I**mplementation is the means by which an intervention is integrated into an organization [25]. To implement complex interventions, usually adaptations are required to ensure a good fit with the individual setting and the staff members who are supposed to apply the intervention in routine clinical care. For this, interventions can be conceptualized as having ‘core components’ (the essential and indispensable elements of the intervention) and an ‘adaptable periphery’ (adaptable elements, structures, and systems related to the intervention and organization into which it is being implemented) [25]. With the preceding two pilot studies ANtiPain has been closely adapted to the German speaking context. While the core components were defined and maintained (i.e., information, skills building, nurse coaching), current context health system factors were taken into account for implementation.

*Standard care* refers to cancer pain management during hospitalization and follow-up according to local standards and international guidelines (e.g., [26]). Standard care was assessed at the start of the study by structured interviews with each ward’s head or designated intervention nurses. In our study, patients did not routinely receive standardized nursing support of pain self-management before implementation of ANtiPain. In one hospital (H3 Table 1), an institutional pain management improvement project was already in place. However, in this project routine pain assessment, documentation and pain medication were addressed but not structured pain self-management support. Therefore, ANtiPain was viewed as an ideal supplement.

**Table 1:** Participating hospitals and wards and recruitment (ITT [PP])

Hospital	Ward	Order^**a**^	Main medical field	% oncology patients^**b**^	size^**c**^	N IG ITT (PP)^**d**^	N CG ITT (PP)^**e**^
H 1	W 9^f^	8	Gynecology	40	25	0	3
H 1	W 10	3	Oncology/Internal medicine	70	21	3	1
H 1	W 11	14	Internal Medicine/Oncology	40	20	0	6
H 1	W 12	1	Pneumology	45	22	3	1
H 1	W 13	12	Pneumology	40	21	0	1
H 2	W 14	17	Internal Medicine/Oncology	60	27	0	5
H 2	W 15	9	Internal Medicine/Oncology	50	27	0	3
H 2	W 16	10	Internal Medicine/Oncology	50	27	0	5
H 2	W 17	2	Oncology/Hematology	98	13	4 (3)	2 (3)
H 3	W 1	15	Surgery	2^g^	32	0	1
H 3	W 2	5	Surgery	70	23	9	4
H 3	W 3	16	Oncology	99	25	0	17
H 3	W 4	11	Gynecology	95	20	8 (6)	21 (23)
H 3	W 5	13	Ear nose and throat	30	24	1	3
H 3	W 6	6	Ear nose and throat	30	24	0	6
H 3	W 7	7	Radiotherapy	98	36	16 (11)	9 (14)
H 3	W 8	4	Radiotherapy	99	12	17 (9)	4 (12)

^a^Order of implementation, each step/sequence between implementations was ~ 24 days

^b^This assumed rate was assessed in an interview with head nurses prior to recruitment of each ward

^c^Number of beds

^d^IG: intervention group, ITT: intention to treat approach - analyzing each patient after implementation of the intervention (PP: per protocol approach - analyzing each patient who received the intervention n in brackets)

^e^CG: control group, ITT: intention to treat approach - analyzing each patient before implementation of the intervention (PP: per protocol approach - analyzing each patient who did not receive the intervention n in brackets)

^f^After recruitment and randomization, an organizational change caused that this ward did not have many oncology patients any more. At inclusion, this ward would have had 50% oncology patients

*ANtiPain* [15, 16] and the original PRO-Self© Plus PCP [14] are based on the Theory of Symptom Management [27] and Bandura’s Social Cognitive Theory [28]. We assume that it reduces barriers and thus changes pain self-management-related behavior leading to a reduction of pain interference with daily activities [16, 18]. In addition, we assumed that the practical aspects of pain management (e.g., timing of analgesic medication in daily routine) would improve patient-related outcomes. A more detailed description of the intervention can be found elsewhere [16, 18]. In short, ANtiPain entails structured (e.g., each patient is taught how to communicate their pain to health care providers) and tailored components. In the tailored part, first, an individualized pain medication plan was set up that was based on the individual analgesic prescription. In a discussion with the patient, a realistic application plan was written down (e.g., which exact time points were ideal in terms of pharmacodynamics in agreement with the patients daily routine and pain trajectory). Second, high patient-related barriers towards pain self-management were addressed using the ‘academic detailing approach’. ‘Academic detailing’ uses patients’ answers to the German version of the Barriers Questionnaire II (BQII-G) to tailor the discussion [14, 29]. As mentioned before, ANtiPain’s structure is adjustable to clinical settings. In this study, designated intervention nurses provided patients and their caring relatives with a face-to-face consultation during hospitalization shortly before discharge. After discharge, one to three phone calls were offered, which followed a clinical algorithm, based on pain intensity, satisfaction with pain management and adherence. As ANtiPain’s core components were maintained, consultations focused on pain assessment, the individual analgesic prescription, side effects, and patient-related barriers. As in the original PRO-Self© PCP studies, patient-related barriers to cancer pain management were addressed with ‘academic detailing’ [14, 29]. In addition, patients received a corresponding booklet, the individualized medication plan, and a numeric pain scale. Intervention nurses followed an intervention protocol, applied assessment instruments (e.g., to assess pain or barriers to pain management [30]), and were asked to use a pocket booklet about common analgesics. Corresponding laminated theme-cards were used to visualize topics for patients covered in the discussion [16, 18]. During the follow-up calls, pain, side effects of the prescribed analgesic medication, as well as adherence to analgesics and given recommendations were assessed and re-discussed.

*Implementation:* For training, each designated intervention nurse received a 1.5-h training session, detailed teaching materials and a case-based coaching throughout the study by the last author (AK). Patient cases were reviewed randomly at each ward after implementation to check for protocol adherence. If deviations from protocols were found, they were taken as cases during the coaching sessions. Results according to the **A**doption domain of the RE-AIM framework will be reported elsewhere.

### Sample and setting

Hospitals were chosen as study center if they had an oncology focus and were willing to implement ANtiPain on wards treating at least 20% oncology patients. As a result, 17 wards of three Viennese general main hospitals (hospital 1, 5 wards; hospital 2, 4 wards; hospital 3, 8 wards) consented to participate. On each ward, 1 to 4 nurses were chosen to complete the intervention. Nurses were asked to become an intervention nurse if they had more than 2 years of experience with oncology patients, were skilled according to the ward nurses and agreed to participate in the study. Intervention nurses were given time to integrate the intervention in their daily routine without financial reimbursement. Patients were eligible if they were over 18 years old, had cancer-related pain ≥3 within the last 2 weeks on an 11-point numeric rating scale, or a regular cancer pain medication and a necessity to practice pain-self-management after discharge. Patients were excluded if they had cognitive, linguistic, emotional, or physical problems that would hamper study participation.

Sample size calculations were performed based on simulations using information from previous pilot studies. We considered 17 wards and 19 study periods (17 intervention steps and one before and one after data collection period). The planned study duration was 454 days (Jan 17 to March 18) divided by 19 study periods resulting in 24 days per study period. For each combination of study period and ward (from now on called “cell”) we expected one patient to be recruited resulting in a sample size of 17 × 19 = 323. A mixed model was assumed for the primary outcome pain interference with daily activities at T2 with intervention (yes/no) as fixed effect and the ward as random effect, where different intraclass correlation coefficients were simulated. Additionally, a potential effect of the study period was considered as a nuisance parameter in the simulations. Assuming an intraclass coefficient of ρ = 0.1, a sample size of *n* = 323 would allow to detect an effect of 0.6 standard deviations at a significance level of α = 0.05 with a power of 90%. For larger intraclass coefficients the power slightly decreases, but even for ρ = 0.4 it was still larger than 80% for an effect of 0.6 standard deviations [45].

### Variables and measurements

*Recruitment rates* were calculated to assess how well the target audience was identified and accessed on the participating wards (**R**each). Demographic data and group comparisons for those who completed the study versus those who dropped out completed the **R**each analysis. Patient-related outcomes were chosen in accordance with the Theory of Symptom Management (**E**ffectiveness) [16, 27]. The primary patient-related outcome was *pain interference with daily activities*. Secondary patient-related outcomes were *pain intensity*, *patient-related barriers towards pain management*, *self-efficacy*, and *health-related quality of life (HRQoL)*. Socio-demographic and clinical characteristics of patients were included as covariates. Data on *dose and timing of intervention* were collected to estimate the degree of implementation of ANtiPain (**I**mplementation). As a classical outcome for implementation research, patient satisfaction was assessed. To reduce patient burden, generic questions and short forms were preferred whenever possible. Data on **I**mplementation were used to evaluate **E**ffectiveness in view of implementation processes.

*Pain interference with daily activities* was assessed with the interference scale of the Brief Pain Inventory (BPI), which is composed of 7 items on 11-point numeric rating scales (NRS; 0 = “no interference” to 10 = “complete interference”) [31]. The BPI interference scale has shown a high internal consistency (Crohnbach’s α = .88 [18], which was confirmed in this study (α = .84 at baseline). *Worst and average pain intensity* were also rated on an 11-point NRS (0 = “no pain” to 10=“worst imaginable pain”) of the BPI [31]. *Patient-related barriers to cancer pain management* were assessed with the German Barriers Questionnaire II short form (BQII-G12) that consists of 12 items scored on 6-point Likert scales (0=“do not agree at all” to 5=“agree very much”) [30]. The BQII-G12 has shown a high internal consistency (α = .83), which was confirmed in this study (α = .82 at baseline) [30, 32, 33]. *Pain-related self-efficacy* was assessed with the German Pain Self-efficacy Questionnaire (FESS) that consists of 10 items scored on 7-point NRS (0 = “very uncertain” to 6 = “very certain”). The internal consistency was high (α = .93) [34], which was also confirmed in this study (α = .89 at baseline). *HRQol* was measured with 2-items scored on 7-point NRS (1 = “very poor” to 7 = “excellent”): A generic question on the overall health status and a generic question on perceived overall quality of life. These questions were derived from the EORTC-QLQ C30 [35]. Two weeks after discharge (T1), patients were asked to rate their satisfaction with (a) the pain self-management support they received in hospital and (b) their overall satisfaction with pain management in hospital on 5-point Lickert scales (1 = “very satisfied”; 2 = “satisfied”; 3 = “not sure”; 4 = “dissatisfied”; 5 = “very dissatisfied”).

*Covariates* like functional status and depression were assessed with the German Eastern Cooperative Oncology Group Performance Status (ECOG-PS) [36, 37] and the Patient Health Questionnaire (PHQ-2) [38]. Both instruments have adequate psychometric properties.

*Organization-related data* included the *recruitment rate* (patients who consented divided by patients who were asked to participate); number of intervention trainings, how many nurses were trained, how many trained nurses actually performed the intervention, *intervention completion rate* (patients who received the intervention divided by the number of patients who were recruited in the intervention period); and intervention dose (i.e., timing, duration).

### Study procedures

According to the stepped wedge design, ANtiPain was implemented into routine oncology care on one ward after another in a 24 days interval (implementation on 17 wards evenly distributed over the planned 15 months study duration; Fig. 1). The order of implementation was determined randomly by a computer-generated list.

Routine patient flows in the departments were not changed in the study. Instead, routinely hospitalized patients were screened for eligibility if they were hospitalized on one of the participating wards. Designated nurses of the participating wards who were supported by study nurses invited eligible patients to attend the study between January 2017 and May 2018, obtained oral and written informed consent by the patients, and performed baseline data collection. Baseline data (T0) were collected prior to discharge and before the face-to-face session in those wards who already implemented ANtiPain. Patient-related follow-up data were collected 2 weeks (T1), 4 weeks (T2), and 8 weeks (T3) after discharge. Patients completed a self-report questionnaire either in paper format or via a corresponding online questionnaire. In addition, the intervention nurses collected clinical and those demographic data that could be derived from the patient records at baseline. Study nurses who collected all data for T1-T3 via post or online questionnaires were blinded to group allocation. Paper questionnaires were stored at the participating clinics and the research institute in locked cabinets, separately from patients’ consent forms and electronic forms on a secured university server. The ethical board of the Viennese Medical University approved the study (1911/2016).

### Data analysis

All data were entered into a password-protected electronic databank. To detect entry failures, 10% of the questionnaires were double entered, yielding an error rate of < 0.1%. When calculating total scores, missing values were replaced by the observed means of the other items. Other missing values and dropouts were not replaced. The main analyses followed the intent-to-treat maxim and was performed for all 17 wards (overall effect), for the wards that recruited patients in the control as well as in the intervention period, and for those wards that recruited at least 10 patients, respectively (implementation effect on effectiveness). For the primary endpoint “pain interference with daily activities”, an additional per-protocol analyses was performed. To analyze the longitudinal data at four time points (T0-T3) linear mixed models were applied with a random intercept for the ward and both a random intercept and a random slope for each individual. Fixed effects included the intervention status (measured binary [yes/no]), actual time passed since T0 and the interaction between intervention status and time, where the interaction term (difference in slopes between patients before intervention and after intervention) was of primary interest. Hypotheses were tested at a significance level of α ≤ .05. The analysis of a stepped wedge design often includes a time trend, but this was not possible in our case due to the uneven distribution of observations over clusters by time and the lack of control patients in 9 wards.

## Results

### Setting and sample

Characteristics of the participating wards (*N* = 17) are displayed in Table 1. Participating wards were located in three hospitals and included a variety of medical fields representing a standard mixture of eligible wards in most general hospitals. In ward 1, major structural changes directly after randomization resulted in a low rate of oncology patients of 2%. Ward sizes ranged from 13 to 36 beds.

**Fig. 2:**
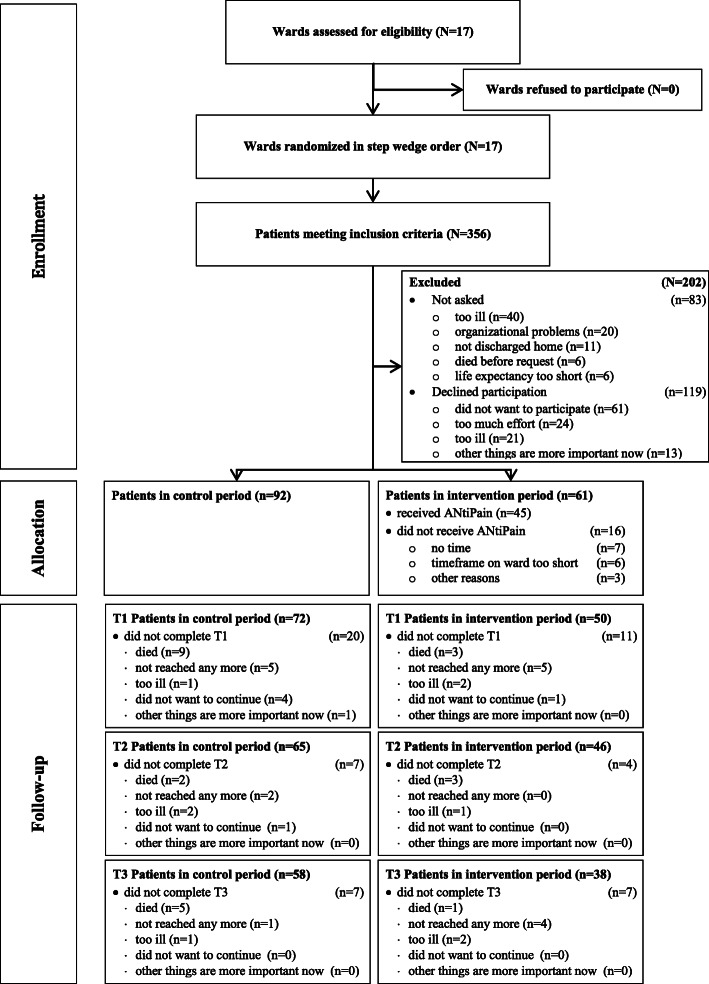
Flow of participants during study

Figure 2 gives an overview of recruitment, dropout, and allocation to control or intervention period. Of the 356 patients who met the inclusion criteria, 83 (23%) were not asked to participate. Most of these 83 eligible patients seemed too ill (48%, *n* = 40) or were not asked due to organizational reasons (i.e., the time, that patients stayed on the ward was too short for recruitment [*n* = 6], lack of time resources of personnel [*n* = 2], or other organizational issues [*n* = 12]). Of the 273 patients who were asked, 153 consented to participate, resulting in a *recruitment rate* of 56% and representing only 50% of the desired 323 patients (Fig. 2).

Recruitment was unevenly distributed over wards and hospitals (Fig. 1; Table 1). In hospital 3 that provided 8 of the 17 study wards (47%), *n* = 116 (76%) patients were recruited, the majority on 5 of these 8 wards (*n* = 105, 68%). This means that 29% of the wards, all within one hospital, recruited 68% of all participants. In contrast, on 9 wards (53%), no patients were recruited in the intervention period (Table 1). The number of beds was not significantly correlated with recruitment numbers, but the estimated rate of oncology patients on the participating wards was (Spearman-rho = .708; *p* = .001). Despite a constant recruitment rate over time (see Figure 5 supplemental material), the uneven recruitment on the wards resulted in a larger number of patients in the control period (Fig. 1; *n* = 92; 60%). In addition, at T2, *n* = 42 patients had dropped out of the study (dropout rate T2 = 27%) while *n* = 96 (63%) patients provided complete datasets (Fig. 2). Patients who dropped out had a lower performance status, higher depressive screening score, less self-efficacy, lower HrQoL, and took more morphine (supplementary Table 1, additional online material). The percentage of patients who dropped out of the intervention group (IG; 41%) did not differ from that in the control group (CG; 42%).

Unfortunately, the number of patients included in the study turned out to be substantially smaller than initially planned. Therefore, implementation was paused for 2 months during summer and the data collection period was prolonged for 2 months at the end. Pausing implementation in summer was chosen for two reasons: (1) summer did not seem to be an ideal time for implementation because of summer holidays; and (2) placing the break in the middle of the recruitment time should theoretically not have resulted in an uneven distribution between control and intervention period (Fig. 1). The study extension resulted in 357 cells (cell_XY_ = cluster X by 24 days step Y; Fig. 1). Still, the number of patients was lower than expected. In 242 (68%) of the 357 cells, no patient could be recruited. To deal with the resulting substantial loss of power compared with the originally planned study our primary statistical analysis was changed. Instead of analyzing specifically differences of outcome variables between groups at time T2 we considered all four time points in the linear mixed model described above and analyzed the difference in slopes between groups.

*Implementation:* Median time between the implementation sequences was as planned 24 days (minimum 20 days, maximum 28 days not counting the 79 days summer break). In total, 35 intervention nurses were trained within 19 training sessions. Median time for training was as planned 1h36min (range: 1h15min to 2 h).

The *intervention completion rate* was 74% (Fig. 2) which means that out of 61 patients in the intervention period, 16 (26%) did not receive the intervention. The most frequent reasons for not giving the intervention were “no time” (11%, *n* = 7) and “patient stay on the ward was too short” (10%, *n* = 6). Other reasons for not providing patients with the intervention included “spontaneous discharge”, “discharge to palliative care unit”, or “intervention nurse was not informed of discharge”. Of the 35 trained intervention nurses, 16 (46%) actually performed at least one intervention. The most frequent reason for nurses not performing any intervention was the lack of recruitment after the training was performed (14 [40%] intervention nurses). In total, 45 interventions were performed entailing a face-to-face session and a median of 2 (range: 0 to 4) phone calls. Mean duration of interventions was 33 min (range: 10 to 95). Mean duration of phone calls was 17 min (range: 1 to 37).

*Demographic and clinical characteristics* at baseline are displayed in Table 2. Despite the random allocation of intervention times to the different wards, the primary outcome of pain intensity differed between the two groups at baseline with patients in the intervention period having slightly lower pain scores compared to those in the control group (Table 2). We have no explanation for this difference at baseline, but our mixed model analysis implicitly accounted for baseline differences between wards. In addition, patients in the intervention period took slightly less strong opioids and more weak opioids compared to patients in the control period. With respect to all other covariates no substantial difference between the two groups were observed.

**Table 2:** Demographic and clinical characteristics of participants

	Control group (*n* = 92)	Intervention group (*n* = 61)
age		
Mean (median); percentile 25/75	58.9 (60.0); 49/73	58.6 (59.0); 52/68
Gender; % (n)		
male	43 (40)	49 (30)
female	57 (52)	51 (31)
Live alone in household; % (n)		
yes	35 (32)	36 (22)
School education; % (n)		
Basic school education	7 (6)	17 (10)
Higher school education/job training	78 (71)	67 (40)
University education	15 (14)	17 (10)
Months since diagnosis		
Mean (median); percentile 25/75	27.4 (6.0); 2/31	17.4 (3.0); 1/11
Diagnosis; % (n)		
Gynecological	24 (22)	16 (10)
Gastrointestinal	22 (20)	20 (12)
Ear nose and throat	13 (12)	23 (14)
LungCa	11 (10)	15 (9)
Hematological	7 (6)	3 (2)
BoneCa	7 (6)	2 (1)
BreastCa	5 (5)	8 (5)
Other (prostate, skin, thoracic, etc)	10 (9)	13 (8)
Missing	2 (2)	0
Painduration in months		
Mean (median); percentile 25/75	20.6 (2.0); 1/9	5.0 (2.0); 0/5
Pain pattern; % (n)		
Constant pain, minor fluctuations	34 (31)	25 (15)
Constant pain, major fluctuations	33 (30)	41 (24)
No constant pain but pain attacks	32 (29)	34 (20)
Performance status (ECOG)		
Mean (median); Percentile 25/75	2.4 (3.0); 2/3	2.2 (2.0); 2/3
PHQ-2		
Mean (median); Percentile 25/75	2.9 (3.0); 2/4	2.6 (2.5); 2/4
Questionnaires completed; % (n)^a^		
Baseline only	20 (18)	15 (9)
Baseline and T2^b^	70 (64)	74 (45)
Complete datasets	59 (54)	57 (35)
BPI pain interference total score T0		
Mean (median); Percentile 25/75	5.6 (6); 4/7	5.0 (5); 4/6
BPI worst pain T0		
Mean (median); Percentile 25/75	7.9 (8.0); 7/9	7.1 (7.5); 5.5/9
BPI average pain T0		
Mean (median); Percentile 25/75	5.9 (6); 5/7	5.2 (5.0); 4/6.5
BQIIG12 T0		
Mean (median); Percentile 25/75	2.1 (2); 1.5/3	2.1 (2); 1/2.5
FESS total score T0		
Mean (median); Percentile 25/75	2.4 (2); 1.5/3	2.2 (2); 1/3
Health status T0		
Mean (median); Percentiles 25/75	3.0 (3); 2/4	2.9 (3); 2/4
Quality of life T0		
Mean (median); Percentiles 25/75	3.1 (3); 2/4	2.8 (3); 2/3
Analgesic medication; % (n)^c^		
No analgesics	2 (2)	2 (1)
Non-opioids	23 (21)	20 (12)
Weak opioids	6 (5)	30 (18)
Strong opioids	69 (63)	48 (29)
Co-Analgesics; % (n)		
yes	28 (25)	17 (10)
Medication schedule; % (n)		
No pain medication	2 (2)	0
Fixed and as needed analgesics	77 (70)	82 (49)
Only fixed scheduled analgesic	14 (13)	13 (8)
Only as needed analgesics	7 (6)	5 (3)
Daily morphine equivalent		
Mean (median); Percentile 25/75	62.3 (40); 0/94	43.8 (20); 0/63
Inadequate analgesia according to PMI; % (n)^d^		
yes	26 (23)	20 (12)

*Abbreviations*: *BPI* brief pain inventory, *BQIIG12* Barriers questionnaire II German - short version, *FESS* Pain related self-efficacy score-German, *ECOG* Eastern Co-operative Oncology Group performance status, *PHQ-2* patient health questionnaire (2-item version)

^a^Percentages do not sum up to 100 because of overlap between the categories; ^b^ T2 was measured 4 weeks after discharge and is the primary measurement time point;); ^c^ according to the WHO step ladder; ^d^ A negative pain management Index indicates inadequate pain

### Primary outcome

The mixed model to analyze the primary outcome included a random intercept and a random slope for each individual and a random intercept for each ward. Two alternative models were fitted, one including additionally a random slope for each ward and the other one discarding the random effect for ward. According to the Bayesian Information Criterion these two models had a slightly worse model fit than the model we used. Furthermore, the exact choice of the random effect for ward had hardly any effect on test results for the fixed effects.

**Fig. 3:**
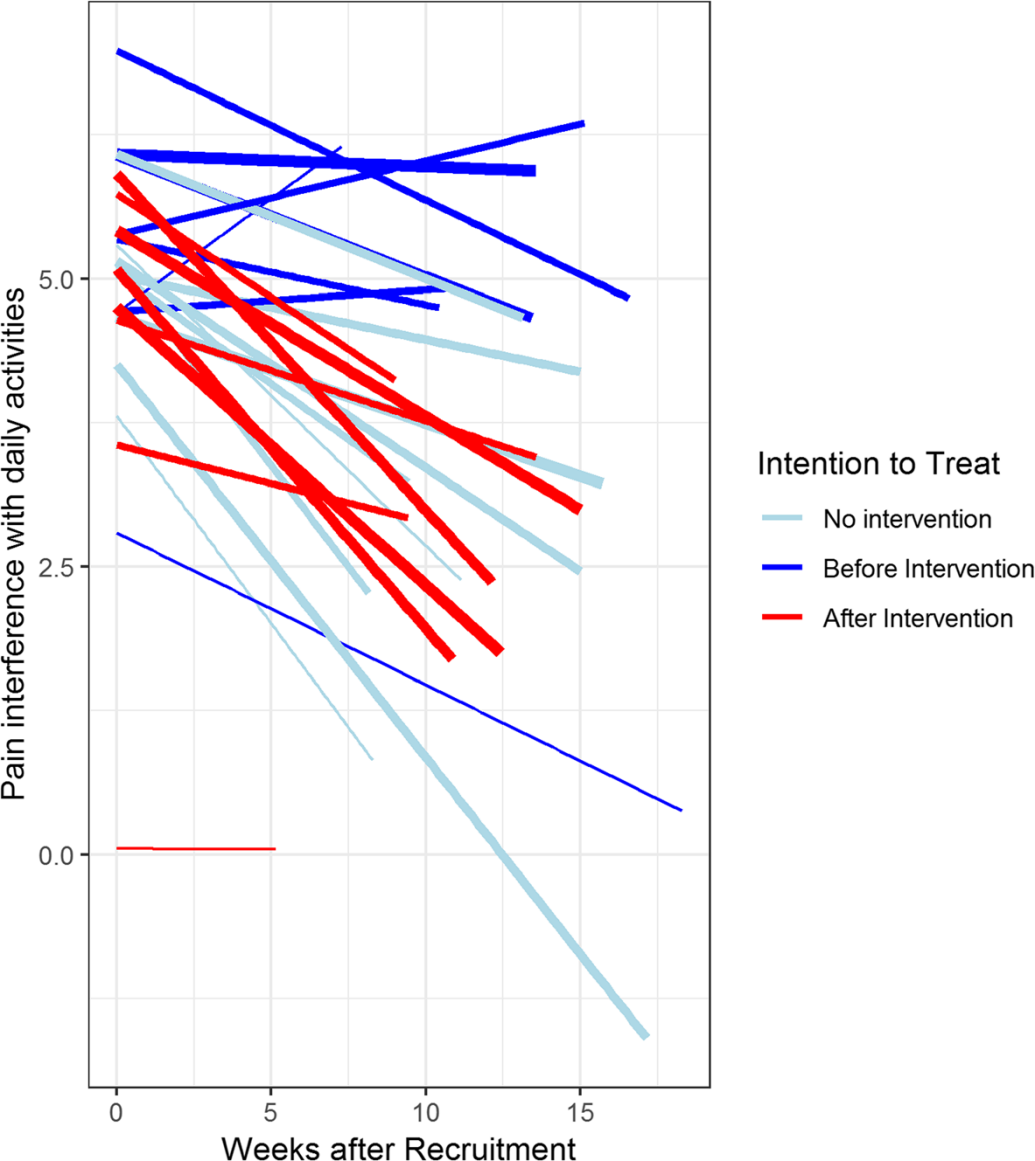
Regression lines of pain interference with daily activities (time in weeks). Each line represents the regression line of one ward per recruitment period. The light blue lines belong to the 9 wards that did not provide any patients in the intervention period. Line thickness represents the number of patients for the respective ward

Following the intention to treat maxim there was no significant difference between the slopes of the two groups (*p* = .198). However, the group-by-time effect was significant when analyzing only the eight wards that provided patients before and after the intervention (*p* = .009). Figure 3 shows estimated regression lines of the primary outcome for wards before intervention (blue) and after intervention (red) where the thickness of the lines corresponds to the number of patients involved. The light blue lines belong to the 9 wards which did not provide any patients in the IG. It is apparent that the decrease in pain in those wards is closer to the decrease in the other 8 wards after intervention (red lines) than before intervention (dark blue lines). This is the reason why we obtain a significant difference between groups when we only consider the 8 wards for which patients with intervention were provided, while there is no significant difference when including all wards. The difference was also significant when analyzing those 5 wards that recruited more than 10 patients (*p* = .0497). No center effect was observed but bearing in mind that the number of observations in two of the three hospitals was quite small (Table 1) this does not come as a surprise.

When performing per-protocol analysis the observed effects of the intervention were systematically larger than for intention-to-treat analysis. The group-by-time effect was still not significant when considering all 17 wards (*p* = .100), whereas smaller *p*-values were obtained for the eight wards with intervention patients (*p* = .011) and the 5 wards that recruited more than 10 patients (*p* = .040).

### Secondary outcomes

**Fig. 4:**
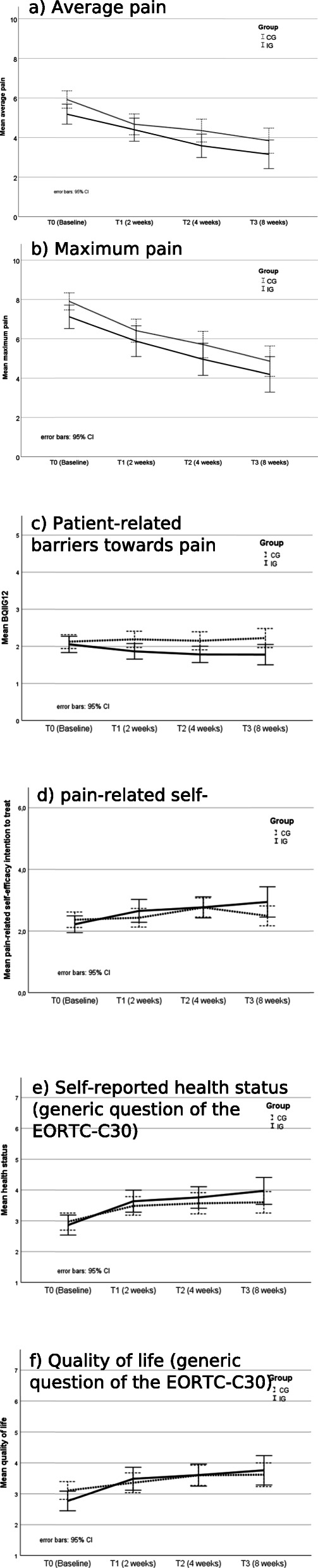
Line diagrams on original scale of secondary outcomes

Line diagrams for secondary outcomes are shown in Fig. 4 and corresponding effect sizes for all outcomes at specific time points are displayed in Table 3. Applying intent-to-treat analysis, the group-by-time effect was significant for self-efficacy (*p* = .033) and for self-reported health status (*p* = .037); for patient-related barriers towards pain management it was just not significant (*p* = .057). Two weeks after discharge, patients in the IG were significantly more satisfied with pain self-management support (IG mean = 2.0, CG mean = 2.2; *p* = .002); and with pain management in hospital (IG mean = 1.8, CG mean = 2.2; *p* = .018).

**Table 3:** Effect sizes of primary and secondary endpoints (intention to treat approach)

Outcome	Cohen’s d T1	Cohen’s d^a^ T2	Cohen’s d T3
Pain interference with daily activities^b^	.12	−.21	−.29
Pain interference with daily activities wards with IG^b^	−.21	−.59	−.54
Average pain^b^	.26	−.04	−.11
Average pain wards with IG^b^	.02	−.27	−.32
Maximum pain^b^	.12	−.02	−.05
Maximum pain wards with IG^b^	.08	−.26	−.23
Barriers towards pain management (BQII-G12)^b^	−.30	−.34	−.46
Barriers towards pain management with IG^b^	−.33	−.31	−.55
Pain-related self-efficacy (FESS)^c^	.25	.17	.53
Pain-related self-efficacy wards with IG^c^	.57	.48	.72
EORTC health status generic question^c^	.16	.26	.37
EORTC health status generic question with IG^c^	.18	.28	.48
EORTC health-related quality of life generic question^c^	.32	.20	.27
EORTC health-related quality of life generic question with IG^c^	.47	.24	.48
Patient satisfaction with pain self-management support^c^	.61	nm	nm
Patient satisfaction with pain self-management support wards with IG^c^	.65	nm	nm
Patient satisfaction with pain management^c^	.46	nm	nm
Patient satisfaction with pain management wards with IG^c^	.62	nm	nm

T1: 2 weeks after discharge; T2: 4 weeks after discharge (primary endpoint printed bold); T3: 8 weeks after discharge; IG: Intervention group; wards with IG: Ward 2, 4, 5, 7, 8, 10, 12 and 17; nm: not measured;

^a^Cohen’s d: .2 < d < .5 small effect, .5 ≤ d < .8 moderate effect, d ≥ .8 large effect; ^b^ Reduction (negative d) desired; ^c^ Rise (positive d) desired;

## Discussion

To our knowledge, this was the first study to evaluate aspects of implementation of a pain self-management support intervention in clinical practice according to the RE-AIM framework. In our study, we could observe statistically and clinically significant effects for those wards that had the chance to apply ANtiPain on a regular basis. This indicates, that the implementation of Antipain seems worthwhile under certain conditions. Careful consideration should be given to the settings, in which a routine application of ANtiPain may be reasonable. In our study, an estimated high proportion of oncology patients routinely treated on the wards was a prerequisite for the application of ANtiPain. Spending much effort in training and coaching intervention nurses in settings with only little proportions of oncology patients seems pointless. However, finding solutions for those patients with cancer-related pain who are hospitalized on wards with only little proportions of oncology patients may still be necessary. In our study, engaging intervention nurses from outside the wards’ teams to provide ANtiPain was limited and largely depending on the time patients stayed on the wards and on discharge procedures. The large proportion of patients who did not receive ANtiPain (e.g., because the time window was too short, or the intervention nurse was not informed of the sudden discharge) shows this.

Our study represents effects one may achieve with a supplementary patient-education approach such as ANtiPain. For institutions that take the challenge to improve patient-related outcomes regarding cancer pain management, ANtiPain may be viewed as one step in a circular quality improvement approach [39]. By a circular approach, multifaceted aspects of pain management can be addressed without overloading the practice setting with too many innovations at once. Still, the application of ANtiPain can reveal other aspects of pain management that may offer room for improvement like for example the process-oriented improvement of pain prescriptions [40]. This will help to observe and synchronize other processes that may be necessary to optimally manage cancer pain in a team approach [39]. In our study, we used a top-down approach to implement pain self-management support in clinical practice. Instead, when practitioners themselves are given the chance to initiate change in a bottom-up approach, this may increase the chances that process changes are adopted in routine care. Most probably, the introduction of pain assessment, documentation and state-of-the-art guidelines for pain management as done in hospital 3 may be a suitable first step before turning to pain self-management support.

One of the most prominent limitations of our study was the uneven distribution of recruitment. Unfortunately, the stepped wedge design does not allow for unforeseeable changes that may happen in clinical settings such as the structural change that cause the low rate of oncology patients on ward 1 [24, 41]. In our analysis, we adjusted for these irregularities. However, we could no longer make use of the advantages of the stepped wedge design (e.g., each ward can act as their own control, time trends may reveal routine uptake) [24, 41]. Another limitation was that the study may be underpowered as recruitment was slower than expected. We tried to compensate for that by performing longitudinal analyses, which considers all available data at four different time points.

Furthermore, baseline differences regarding pain intensity were observed. On the one hand, these baseline difference may be due to between cluster differences, e.g., ward 3 added 17 (11%) patients only in the control period with a baseline pain score clearly higher than the other wards (mean max pain = 8.2 versus 7.5). On the other hand, baseline differences may be due to within cluster differences. In the analysis, we accounted for these differences.

In our study, implementation was initiated from outside the institutions. We highly recommend embedding implementation in a larger scale project of pain management improvement because patient-related barriers only represent “one side of the medal” [42]. In hospital 3, an overarching project to improve pain management, targeting more than the nursing profession, was already in place and ANtiPain was taken on as a supplementary measure. As a result, recruitment and implementation were more successful in this hospital. In a cyclic quality improvement project, interventions may follow the initial implementation of ANtiPain, in which routine application and **A**doption are tested and supported [39].

Surprisingly, effects on patient-related barriers to pain management were small and non-significant even though in the pilot studies these effects already were significant despite the small sample size. Even though still in the range of protocol adherence, protocol performance in EvANtiPain differed from that in the pilot studies [15, 16]. As the mean duration of the interventions in the pilot study (ANtiPain) was nearly double to that in EvANtiPain (1h07min versus 33 min), one needs to think what was cut short in clinical practice. An analysis of the intervention protocols suggested that the medication plan in which quite practical details of medication intake were addressed (e.g., correct dosing and timing of medication) was preferred over the more “theoretical” ‘academic detailing’ approach in which the BQII-G12 was used to tailor a discussion on patient-related barriers. As a result, patients may have been more able to put pain management into practice at home than to overcome their cognitive barriers.

In terms of resources needed for successful implementation, the 1.5-h training for intervention nurses seems the minimum input intervention nurses need for optimal application of ANtiPain. This short training was only possible because exceptionally skilled intervention nurses were chosen. Probably, the academic detailing approach may be better applied when communicative skills are addressed in an additional training session for the intervention nurses. On the other hand, a reduction of patient-related barriers not necessarily translated directly into improved pain-related outcomes [43]. In our study, oral feedback of some intervention nurses implied that the academic detailing approach may be to “highbrow” or theoretical for lay people. Tailoring, however, seems necessary as it is timesaving and more feasible than a generic approach [44]. Future research may pursue didactically more suitable approaches to tailor discussions on patient-related barriers.

## Conclusions

The good news is: implementation of pain self-management support in hospitalized patients shortly before discharge is worthwhile. However, the implementation is only under specific circumstances reasonable. (1) The pain self-management support program needs to be adaptable to different clinical settings and treatment paths entailing essential core components and a flexible structure. (2) The implementation of self-management support may better be embedded in a larger scale program to improve pain management with a multi-professional focus. (3) A critical proportion of oncology patients of 50 to 70% oncology patients on the ward to achieve a certain level of “routine” seems necessary. Solutions for those wards with less than 50% oncology patients may depend largely on time of stay as well as on discharge and treatment standards.

## Supplementary information

**Additional file 1.** Supplemental material Figure 5: Target-performance recruitment.

**Additional file 2.** Supplementary Table 1: Demographic and clinical characteristics of patients who dropped out versus patients who completed the study.

## Data Availability

The datasets used and/or analyzed during the current study are available from the corresponding author on reasonable request.

## Abbreviations

ANtiPain: First pilot study to test the pain self-management support intervention ANtiPain
BPI: Brief Pain Inventory
BQII-G12: German short form of the Barriers Questionnaire II
CG: Control group
ECOG-PS: German Eastern Cooperative Oncology Group Performance Status
EvANtiPain: Acronym of current study
FESS: German questionnaire to assess pain-related self-efficacy
HRQoL: Health related quality of life
IG: Intervention group
NRS: Numeric rating scale
PEINCA: First pilot study to translate and adapt the PRO-Self© Plus PCP
PHQ-2: Patient health questionnaire (2 items)
PRO-Self© Plus PCP: PRO-Self© Plus Pain Control Program
RCT: Randomized cotrolled clinical trial
RE-AIM: Reach Effectiveness Adoption Implementation and Maintenance
