# Supplementary figures and images for: Implementation of a nurse-led self-management support intervention for patients with cancer-related pain: a cluster randomized phase-IV study with a stepped wedge design (EvANtiPain)
Source: BMC Cancer. 2020 Jun 16;20:559. doi: 10.1186/s12885-020-06729-0 (PMC7296932; doi:10.1186/s12885-020-06729-0)

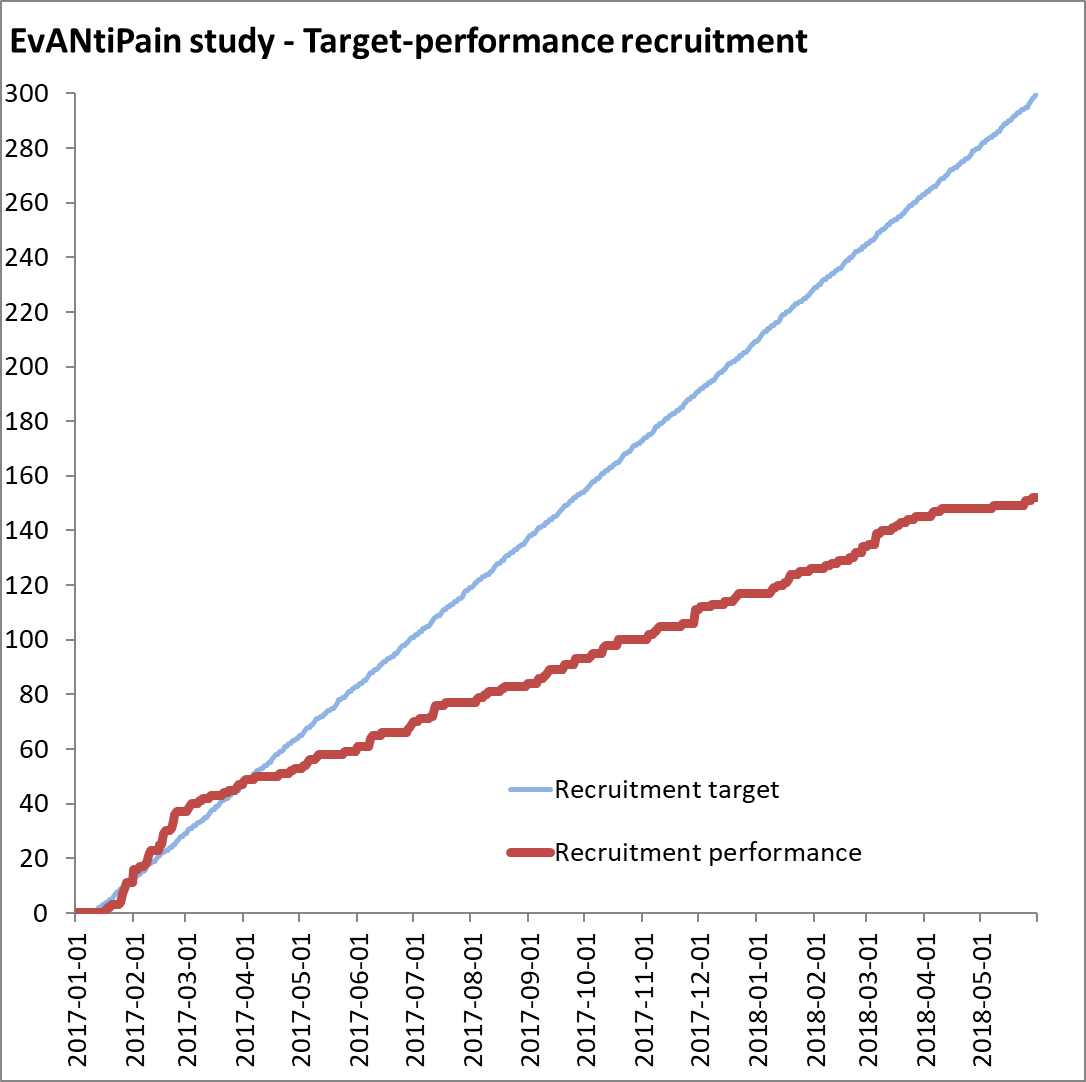

Supplement: Supplementary file 1 — Additional file 1. Supplemental material Figure 5: Target-performance recruitment. [file 12885_2020_6729_MOESM1_ESM.docx]
