# Supplementary material for: Implementation of a nurse-led self-management support intervention for patients with cancer-related pain: a cluster randomized phase-IV study with a stepped wedge design (EvANtiPain)
Source: BMC Cancer. 2020 Jun 16;20:559. doi: 10.1186/s12885-020-06729-0 (PMC7296932; doi:10.1186/s12885-020-06729-0)
Supplement: Supplementary file 2 — Additional file 2. Supplementary Table 1: Demographic and clinical characteristics of patients who dropped out versus patients who completed the study. [file 12885_2020_6729_MOESM2_ESM.docx]

# Additional online material

**Supplementary Table 1:** Demographic and clinical characteristics of patients who dropped out versus patients who completed the study.

|  |  | Drop outs (n=64) | Patients who completed the study (n=89) | p-Werte (unkorrigiert) |
| --- | --- | --- | --- | --- |
| age | Mean (median); percentile 25/75 | 58.2 (58.5); 51/70 | 59.2 (59.0); 51/71 | .713 |
| Gender; % (n) | male | 45 (29) | 46 (41) | .529 |
|  | female | 55 (35) | 54 (48) |  |
| Live alone in household; % (n) | yes | 27 (42) | 27 (31) | .173 |
| School education; % (n) | Basic school education | 6 (10) | 10 (11) | .633 |
|  | Higher school education/job training | 44 (71) | 67 (75) |  |
|  | University education | 12 (19) | 12 (14) |  |
| Months since diagnosis | Mean (median); percentile 25/75 | 25.7 (6.0); 2/30 | 21.7 (4.0); 1/20 | .488 |
| Diagnosis; % (n) | Gynecological | 23 (15) | 19 (17) | .885 |
|  | Gastrointestinal | 17 (11) | 24 (21) |  |
|  | Ear nose and throat | 14 (9) | 19 (17) |  |
|  | LungCa | 14 (9) | 11 (10) |  |
|  | Hematological | 8 (5) | 3 (3) |  |
|  | BoneCa | 5 (3) | 5 (4) |  |
|  | BreastCa | 8 (5) | 8 (5) |  |
|  | Other (prostate, skin, thoracic, etc) | 9 (6) | 12 (11) |  |
|  | Missing | 2 (1) | 2 (1) |  |
| Painduration in months | Mean (median); percentile 25/75 | 23.7 (2.0); 0/7 | 7.9 (2.0); 1/8 | .347 |
| Pain pattern; % (n) | Constant pain, minor fluctuations |  |  | .438 |
|  | Constant pain, major fluctuations |  |  |  |
|  | No constant pain but pain attacks |  |  |  |
| Performance status (ECOG) | Mean (median); Percentile 25/75 | 2.6 (3.0); 2/3 | 2.1 (2.0); 1/3 | .002* |
| PHQ-2 | Mean (median); Percentile 25/75 | 3.1 (3.0); 2/4 | 2.6 (2.0); 2/4 | .034* |
| BPI pain interference total score T0 | Mean (median); Percentile 25/75 | 5.9 (6); 4/8 | 5.0 (5); 4/6 | .010* |
| BPI worst pain T0 | Mean (median); Percentile 25/75 | 7.9 (8.0); 7/10 | 7.4 (8.0); 6/9 | .123 |
| BPI average pain T0 | Mean (median); Percentile 25/75 | 5.8 (6); 4/7 | 5.5 (5.0); 4/7 | .350 |
| BQIIG12 T0 | Mean (median); Percentile 25/75 | 2.0 (2); 2/3 | 2.2 (2.3); 2/3 | .294 |
| FESS total score T0 | Mean (median); Percentile 25/75 | 2.1 (1.9); 1/3 | 2.5 (2.5); 2/3 | .028* |
| Health status T0 | Mean (median); Percentiles 25/75 | 2.7 (3); 2/4 | 3.1 (3); 2/4 | .133 |
| Quality of life T0 | Mean (median); Percentiles 25/75 | 2.7 (3); 2/4 | 3.2 (3); 2/4 | .010* |
| Analgesic medication; % (n)^c^ | No analgesics |  |  | .321 |
|  | Non-opioids |  |  |  |
|  | Weak opioids |  |  |  |
|  | Strong opioids |  |  |  |
| Co-Analgesics; % (n) | yes | 27 (17) | 20 (18) | .331 |
| Medication schedule; % (n) | No pain medication |  |  | .167 |
|  | Fixed and as needed analgesics |  |  |  |
|  | Only fixed scheduled analgesic |  |  |  |
|  | Only as needed analgesics |  |  |  |
| Daily morphine equivalent | Mean (median); Percentile 25/75 | 61.4 (45); 15/95 | 41.0 (28.8); 0/63 | .022* |
| Inadequate analgesia according to PMI; % (n)^d^ | yes | 18 (11) | 27 (24) | .241 |
